# Neurocognitive findings in young adults with binge eating disorder

**DOI:** 10.1080/13651501.2019.1687724

**Published:** 2019-11-14

**Authors:** Jon E. Grant, Samuel R. Chamberlain

**Affiliations:** a Department of Psychiatry and Behavioral Neuroscience, University of Chicago, Pritzker School of Medicine, Chicago, IL, USA;; b Department of Psychiatry, University of Cambridge, Cambridge, UK;; c Cambridge and Peterborough NHS Foundation Trust, Fulbourn, UK

**Keywords:** Impulsivity, compulsivity, binge, food, addiction

## Abstract

**Objectives:** Binge-eating disorder (BED) has been associated with cognitive impairment, including on measures of impulsivity, but it is not clear in prior literature whether these deficits may have been associated with obesity, rather than BED per se. Impulsivity may play a role in predisposing people towards BED as well as in the chronicity of symptoms. The aim of this study was to examine cognitive functions between BED and healthy controls matched for age, gender, and body mass indices.

**Methods:** Individuals with BED and healthy controls were recruited from the general community using media advertisements. After providing informed consent, study participants completed a clinical interview and computerised neuropsychological testing. Group differences were analysed.

**Results:** Groups did not differ significantly on age, gender, education levels, or body mass indices. The BED group (*N* = 17) exhibited significantly impaired stop-signal response inhibition (Stop-Signal Task) and executive planning (Stockings of Cambridge Task) compared to healthy controls (*N* = 17). Spatial working memory and set-shifting were intact.

**Discussion:** BED appears to be associated with motor disinhibition and impaired executive planning even controlling for obesity. Longitudinal work is needed to clarify whether motor impulsivity predisposes people to BED, and/or contributes to persistence of symptoms over time.

**Key points**
- Binge-eating disorder is common, under-recognised, and associated with untoward physical and health sequelae.
- The neurobiological basis of binge-eating disorder is unclear; cognitive testing may offer insights.
- Many prior cognitive studies have not controlled for potential confounds, especially group differences in body mass indices (BMI). Obesity in itself has been linked with cognitive dysfunction.
- Here, we compared cognition between people with binge-eating disorder and controls, matched for BMI and other measures.
- Binge-eating disorder was associated with impaired response inhibition and executive planning.
- These results inform neurobiological models of binge-eating disorder and may suggest new treatment targets for this condition.

## Introduction

Binge eating disorder (BED), also referred to as pathological overeating, has been reported in the medical literature, in different forms and under various names, for over a century, but was first carefully described as a disorder in 1992 (Stunkard, 1997). Characterised by consuming objectively large amounts of food in discrete time periods while experiencing a sense of ‘loss of control’, BED leads to significant psychosocial dysfunction and represents a major public health concern (American Psychiatric Association, 2013). It is associated with physical health sequelae including increased mortality, risk of type 2 diabetes, hypertension, chronic pain, and headaches (Kessler et al., 2013; Kornstein, Kunovac, Herman, & Culpepper, 2016). Furthermore, the majority of people with lifetime BED develop one or more comorbid mental health diagnoses at some point, especially mood and anxiety disorders (Kessler et al., 2013). In treatment seeking patients with BED, substantially increased rates of other eating disorders, depression, bipolar disorder, anxiety disorders, post-traumatic stress disorder, and suicide attempts, were found (Welch et al., 2016). Recent studies have indicated that approximately 1% to 4.6% of the general population endorses symptoms consistent with BED (Hudson, Hiripi, Pope, & Kessler, 2007). BED is common but often under-reported in medical settings. For example, obese individuals are at 2–3 times increased risk of having disordered eating, compared to normal weight individuals (Nagata, Garber, Tabler, Murray, & Bibbins-Domingo, 2018). In a meta-analysis of the available data, BED was present in 17% of people seeking and undergoing bariatric surgery (Dawes et al., 2016).

Research has suggested that impulsivity may be a core cognitive underpinning of BED (Kaisari, Dourish, Rotshtein, & Higgs, 2018). Impulsivity is a broad term referring to a disposition towards behaviours that are unduly hasty, risky, and that lead to undesirous outcomes (Grant & Chamberlain, 2014). Aspects of impulsivity can be quantified objectively using questionnaires and also cognitive tasks. Several distinct types of impulsivity have been delineated on computerised cognitive tasks including disinhibition of motor responses (such as measured using the Stop-Signal task) and risky decision-making (such as measured using Gambling tasks) (Grant & Chamberlain, 2014).

Impulsivity could relate to BED in several ways: it may predispose people towards a range of behaviours including binge-eating (and consequent weight gain); but may also constitute a perpetuating factor meaning that affected individuals cannot stop the maladaptive binge-eating behaviours once established. In fact various facets of impulsivity have been associated with BED when compared to healthy controls: executive functioning deficits (Boeka & Lokken, 2011; Duchesne et al., 2010); deficits on attention and inhibitory control (using a food/body-mental flexibility task) (Mobbs, Iglesias, Golay, & Van der Linden, 2011); impaired cognitive flexibility (using the Trail Making Test) (Svaldi, Brand, & Tuschen-Caffier, 2010); and reduced preference for delayed rewards (Manwaring, Green, Myerson, Strube, & Wilfley, 2011). However, obesity itself may be associated with cognitive impairment. For example, obese individuals showed elevated impulsivity both on the Stop-Signal task and Barratt impulsiveness questionnaire, compared to normal weight controls with similar levels of impulse control disorders (Chamberlain, Derbyshire, Leppink, & Grant, 2015). Some studies suggest that cognitive deficits in BED are really primarily associated with obesity, not BED, and that obese individuals with or without BED exhibit the same types and severity of cognitive deficits: *viz*, global cognitive functioning (Galioto et al., 2012), decision-making on Iowa Gambling task (Danner, Ouwehand, van Haastert, Hornsveld, & de Ridder, 2012), decision-making on Iowa Gambling task and a delay discounting measure (Davis, Patte, Curtis, & Reid, 2010), and decision-making (Game of Dice task) plus impulse control (Stop-Signal task) (Wu et al., 2013). Furthermore, cognitive findings for BED are inconsistent. In a systematic review, inhibitory dyscontrol in BED was evident for tasks using non-neutral stimuli (e.g., food images) but not for tasks using neutral cues (Kittel, Brauhardt, & Hilbert, 2015). This systematic review also served to highlight the relatively small number of data studies in this neglected area.

In an attempt to better understand the cognitive underpinnings of BED, we sought to eliminate the confounding variables of body mass index as well as age and gender. Thus, we collected data on young adults aged 18–29 years with BED and healthy controls matched on age, gender, and body mass index. We hypothesised that those with BED would exhibit greater levels of impulsivity on standardised measures. To explore the specificity of any impulsivity-related cognitive deficits, we also included tasks of several other core cognitive domains: set-shifting, spatial working memory, and executive planning.

## Materials and methods

Young adults (18–29 years of age) meeting DSM-5 criteria for binge eating disorder (BED), and healthy controls, were recruited from a metropolitan area by media advertisements for a study of impulsivity in young adults. All participants underwent a detailed psychiatric evaluation (described below). Participants were excluded if they were unable to understand or consent to the study procedures. Controls were excluded if they had any identified mental disorders. All study procedures were carried out in accordance with the Declaration of Helsinki. The Institutional Review Boards of the Universities of Chicago approved the study and the consent statements. After a complete description of the study procedures, participants provided written informed consent. Participants were compensated for their time with a $50 gift card.

### Clinical assessments

Trained raters assessed each participant using the Mini-International Neuropsychiatric Interview (DSM-IV version) (Sheehan et al., 1998), which screens for mainstream mental disorders (e.g., depression, anxiety). The binge-eating disorder module from the Minnesota Impulse Disorders Inventory (MIDI) v1.1 was used to identify BED (Chamberlain & Grant, 2018). For BED, presence of anorexia nervosa or bulimia nervosa (MINI) were exclusionary. Participants were also asked about any history of diagnosed psychiatric conditions such as ADHD.

In addition, participants were evaluated using the following instruments: Yale Brown Obsessive Compulsive Scale Modified for Binge Eating Disorder (BED-YBOCS) examining the severity of urges/thoughts of binge eating, binge eating behaviour, and the extent to which both of these interfere with the person’s life and cause distress (Deal, Wirth, Gasior, Herman, & McElroy, 2015); Quality of Life Inventory (QOLI), a 16-item, self-administered measure of life satisfaction across 16 domains thought to contribute to human happiness and contentment (Frisch et al., 2005); Hamilton Depression Rating Scale (HAM-D), a 17-item, clinician-administered scale assessing depressive symptoms (Hamilton, 1960); and the Hamilton Anxiety Rating Scale (HAM-A), a 14-item, clinician-administered scale measuring global anxiety (Hamilton, 1959); and the Sheehan Disability Scale (SDS) a three-item scale of psychosocial impairment (Sheehan, 1983).

### Cognitive assessments

Cognitive testing consisted of previously validated paradigms from the Cambridge Neuropsychological Test Automated Battery (CANTAB) (CANTABeclipse, version 3; Cambridge Cognition Ltd.). The choice of cognitive tasks was based on existing literature regarding cognition in BED. All testing was conducted in the same controlled environment with a fixed order of the tasks. Outcome measures of interest were selected *a priori*, before data analysis, based on the authors’ expertise in neuropsychological assessment; and with a view to minimising the number of multiple comparisons. In addition to two tasks measuring different aspects of impulsivity (Stop-Signal and Cambridge Gamble tasks) we also quantified set-shifting, spatial working memory, and executive planning. Our rationale for including these non-impulsivity tasks was to assess the extent to which any impulsivity-related deficits were specific in terms of the neuropsychological profile of BED. These domains were also included because they are widely regarded as distinct, important cognitive functions in day-to-day life; and are dependent on the integrity of the frontal lobes.

**Intra-dimensional/Extra-dimensional Set-Shift task (IED)** (Owen, Roberts, Polkey, Sahakian, & Robbins, 1991). The IED is a computerised version of the Wisconsin Card Sorting task. Participants are tasked with learning an underlying rule established by the computer, and once the rule is learned, the rule will be switched and the participant must re-learn the new rule. On each trial, two pictures are shown, and the subject selects the picture they believe to be correct. After each choice, feedback is given (‘correct’ or ‘incorrect’ on-screen) to facilitate learning. Outcome measure of interest was total errors adjusted for stages not completed.

**Stop-Signal Task (SST)** (Aron, Robbins, & Poldrack, 2004). The SST measures control over pre-potent (i.e., habitual or dominant) motor behaviour. Subjects view directional arrows (left/right) appearing one at a time, and make corresponding responses on a button box (left button for left arrow, and *vice versa*). On a minority of trials, a stop-signal (auditory beep) occurs, and volunteers attempt to withhold their response on the given trial. The primary outcome measure is the stop-signal reaction time (SSRT), with longer SSRTs indicative of greater difficulties with motor inhibition.

**One Touch Stockings of Cambridge task (OTS)** (Owen et al., 1995). The OTS is a task assessing executive functioning. Presented with two sets of Coloured balls arranged within three different stacks, participants are asked to mentally calculate the minimum number of moves necessary to rearrange the balls in the first set to match the array of balls presented in the second set. Outcome measure of interest was the number of problems solved correctly on the first attempt.

**Cambridge Gamble Task (CGT)** (Clark, Manes, Antoun, Sahakian, & Robbins, 2003). The CGT measures aspects of impulsive choice. On each trial there are ten boxes (a mix of blue and red), and a token is hidden behind one of these. The participant first chooses the colour they believe the token is hidden behind (blue or red) and then makes a decision about what proportion of their accrued points to gamble. If they made the correct colour choice, they gain points; and if not, they lose the equivalent amount of points. Key outcome measures are overall proportion bet, quality of decision-making (proportion of trials when the logical colour choice was made), and risk-adjustment (a measure of the extent to which participants modulate how much they gamble as a function of the risk of loss/reward).

**Spatial Working Memory Task (SWM)** (Owen, Morris, Sahakian, Polkey, & Robbins, 1996). The SWM is a memory task wherein participants must locate a token hidden under one of a few randomly located boxes on the screen. They are told in advance to avoid returning to search in boxes where tokens have already been ‘found’, thereby assessing working memory function (the ability to hold information about spatial locations online). The outcome measure of interest was the total errors made (i.e., revisiting a box more than one time) in searching for the coin.

### Data analysis

Differences in demographic, clinical, and cognitive measures between the study groups were compared using analysis of variance (ANOVA) or suitable non-parametric tests (likelihood ratios, also known as likelihood ratio Chi-Square statistics) as indicated in the results tables. Significance was defined as *p* < .05, two-tailed. This being an exploratory study, and in view of the sample size, there was no correction for multiplicity. All analyses were undertaken using JMP Pro.

## Results

Participants within the BED group binged a mean (standard deviation, SD) of 4.1 (1.3) days each week. The BED-YBOCS average scores were: urge score of 9.6 (2.6), behaviour score of 10.8 (2.3), and total score 20.4 (4.2). The BED group reported subclinical levels of depression and anxiety: HAM-D 2.65 (2.12), HAM-D 1.69 (1.58); mean Sheehan Disability scores of 11.63 (6.35), and mean quality of life scores of 9.25 (28.92). The following comorbidities, identified on the MINI, were present in the BED group (number of patients and [%]): agoraphobia 2 [11.8%], social phobia 1 [5.9%], cannabis use disorder 1 [5.9%], and antisocial personality disorder 1 [5.9%]. There were no cases of anorexia nervosa or bulimia nervosa. None had a history of diagnosed ADHD, and none were taking psychotropic medications. For clinical variables compared to healthy controls, see Table 1.

**Table 1.** Demographic and clinical characteristics of young adults with binge eating disorder (BED).

Variable	Controls (*N* = 17)	BED (*N* = 17)	Statistic	Value	*p* value
Age, years	23.76 (4.09)	25.47 (4.82)	*F* (1,33)	1.238	.274
Gender, male *N* [%]	6 [35.3%]	6 [35.3%]	LR	1	>.999
Education levels	3.12 (0.93)	3.41 (1)	*F* (1,33)	0.787	.382
Body mass index (BMI), kg/m^2^	31.39 (6.28)	33.87 (5.08)	*F* (1,33)	1.597	.216

Statistical tests refer to ANOVA except where indicated ‘LR’ Likelihood Ratio chi-square; *p < .05.

Results from cognitive testing are presented in Table 2. BED participants exhibited significant impairments compared to controls on the SSRT and OTS Problems solved on first choice.

**Table 2.** Cognitive performance of young adults with binge eating disorder (BED).

Variable	Controls (*N* = 17)	BED (*N* = 17)	Statistic	Value	*p* value	Cohen’s D
IED Total errors (adjusted)	23 (17.51)	24.81 (22.3)	*F* (1,32)	0.068	.796	0.07
SST SSRT	178.29 (62.81)	255.1 (122.9)	*F* (1,32)	5.203	.03*	0.81
OTS Problems solved on first choice	16.71 (3.77)	13.5 (4.47)	*F* (1,32)	4.978	.033*	0.79
CGT Overall proportion bet	0.48 (0.1)	0.53 (0.16)	*F* (1,32)	1.142	.294	0.38
CGT Quality of decision making	0.94 (0.06)	0.9 (0.17)	*F* (1,32)	1.042	.315	0.36
CGT Risk adjustment	1.38 (1.3)	1.17 (1.49)	*F* (1,32)	0.188	.668	0.12
SWM Total errors	23.94 (19.44)	37.19 (25.42)	*F* (1,32)	2.848	.102	0.60

IED: intra-dimensional/extra-dimensional set-shift task; SST: stop-signal task; SSRT: stop-signal reaction time; OTS: one touch stockings of Cambridge task; CGT: Cambridge gamble task; SWM: spatial working memory task.

Statistical tests refer to ANOVA.

*p < .05.

## Discussion

The neuropsychological profile of binge-eating disorder (BED) is under-studied. In a review of the available data in 2011 (Van den Eynde et al., 2011), four case-control studies for BED were identified as compared to 37 for bulimia nervosa. This prior study was unable to conduct a meta-analysis, highlighting the frequent use of non-standardised cognitive tasks, rendering pooling of data problematic. In a more recent review of the ∼14 studies in BED, cognitive deficits were described in domains of inhibition, decision-making, and executive functioning, relative to control groups (Kessler, Hutson, Herman, & Potenza, 2016). Here, we used a standardised computerised testing battery in BED and controls who were matched for age, gender, educational levels, and obesity levels (body mass indices). The key findings were that BED was associated with significantly impaired response inhibition (large effect size) and executive planning (large effect size) relative to controls, but not set-shifting, decision-making, or spatial working memory. These findings accord well with a previous adapted Stop-signal study, which found that motor inhibition deficits in binge-eating occur with neutral cues, rather than only tasks using food-related stimuli (Manasse et al., 2016b).

Although people with BED may binge for many different reasons (e.g., emotional regulation, poor coping, loneliness, interpersonal sensitivity, etc.) (Schulz & Laessle, 2012; Solmi et al., 2018; Spada et al., 2016), focussing on neurocognitive deficits as a central characteristic may help identify a common underpinning to many of the different motivations that individuals report as initiating or maintaining their eating behaviour. If the cognitive problems identified in this analysis are actually core feature of BED, this may have notable clinical implications. Rather than focussing on the eating behaviour or the food, it may be more effective to address the underlying problems in neurocognition. Interestingly, the cognitive profile found here in BED may differ from that found in bulimia nervosa, which was characterised by decision-making impairment, but intact response inhibition and visuospatial functioning (Degortes, Tenconi, Santonastaso, & Favaro, 2016).

Several limitations should be considered. This was a young sample, but BED is common in older age groups. The sample size was relatively small, meaning that the study was underpowered to detect significant group differences with small or medium effect sizes, and that we did not correct for multiple comparisons (since such analysis would have been demonstrably under-powered). Thus, it is possible that BED is associated with subtle impairments in the other domains, even though these differences were non-significant herein, particularly for spatial working memory in view of the effect size obtained. The study was neither designed nor powered to examine the influence of comorbidities on the cognitive profile of BED, an issue that warrants further study in future. For example, ADHD is common in BED and has interesting overlap neurobiologically (including in terms of pharmacotherapy) (Cortese, Bernardina, & Mouren, 2008), but none of our sample had a history of this comorbid diagnosis. Another potential limitation is that we excluded mental disorders in controls but not comorbidities in the cases. The current findings may not generalise to clinical settings, since the study recruited from the general population; however, the corollary is that the findings may be more representative of BED in the general community than had we used clinical settings for recruitment. We did not examine all possible cognitive domains that would have been of interest – for example, we did not include tasks of attentional processing bias for food-related stimuli; such tasks tend to be non-standardised in any event. Lastly, we had a strong rationale for matching groups on BMI (to avoid this confound), but this process could theoretically introduce collider-stratification bias if impulsivity and BED both result in higher BMI. Also, we did not include a normal weight control group.

In summary, we found impaired response inhibition and executive planning in BED compared to well-matched controls, with large effect sizes, suggesting these domains are relatively highly affected in this disorder. Some aspects of impulsivity have been found to relate to worse treatment outcomes in binge-eating (Manasse et al., 2016a), hence the current results may have treatment implications. Further research on neurocognition and neurobiology of BED is needed, particularly in order to investigate whether cognitive impulsivity is a predisposing/vulnerability marker for BED; and whether it contributes to maintenance of binge-eating symptoms over time once established. Future work should also examine whether cognitive findings differ between non-treatment-seeking samples (as was the case here), and treatment-seeking samples.
